# Multi-Scale Agent-Based Multiple Myeloma Cancer Modeling and the Related Study of the Balance between Osteoclasts and Osteoblasts

**DOI:** 10.1371/journal.pone.0143206

**Published:** 2015-12-11

**Authors:** Minna Qiao, Dan Wu, Michelle Carey, Xiaobo Zhou, Le Zhang

**Affiliations:** 1 College of Computer and Information Science, Southwest University, Chongqing, P. R. China; 2 Department of Radiology, Wake Forest University School of Medicine, Winston Salem, United States of America; 3 Department of Biostatistics and Computational Biology, University of Rochester, Rochester, United States of America; Beijing Institute of Genomics, Chinese Academy of Sciences, CHINA

## Abstract

**Research Background:**

Currently, multiple myeloma is the second most common hematological malignancy in the U.S., constituting 1% of all cancers. With conventional treatment, the median survival time is typically 3–4 years, although it can be extended to 5–7 years or longer with advanced treatments. Recent research indicated that an increase in osteoclast (OC) activity is often associated withmultiple myeloma (MM) and that a decrease inosteoblast (OB) activity contributesto the osteolytic lesions in MM. Normally, the populations of OCs and OBs are inequilibrium, and an imbalance in this statecontributes to the development of lesions.

**Research procedures:**

A multi-scale agent-based multiple myeloma model was developed to simulate the proliferation, migration and death of OBs and OCs. Subsequently, this model was employed to investigate the efficacy of thethree most commonly used drugs for MM treatment under the following two premises: the reduction in the progression of MM and the re-establishment of the equilibrium between OCs and OBs.

**Research purposes:**

The simulated results not only demonstrated the capacity of the model to choose optimal combinations of the drugs but also showed that the optimal use of the three drugs can restore the balance between OCs and OBs as well as kill MMs. Furthermore, the drug synergism analysis function of the model revealed that restoring the balance between OBs and OCs can significantly increase the efficacy of drugs against tumor cells.

## Introduction

Previous studies[1] stated that multiple myeloma is the second most common hematological malignancy in the U.S. (after non-Hodgkin lymphoma), constituting 1% of all cancers. Multiple myeloma treatmentcan be classified intothe following three methods. Thefirst is high-dose chemotherapy with autologous hematopoietic stemcell transplantation, which can prolong overall survival and evoke complete remission, but it is not curative. The second is allogeneic stem cell transplantation, which can cure MM in a small percentage of patients with significant side effects [1]. The third is chemotherapy withthe following drug combinations: 1,bortezomib, melphalan, andprednisone, with an estimated overall survival of 83% at 30 months [2]; 2,lenalidomide plus low-dose dexamethasone,with 82% survival at two years[3];and 3,melphalan, prednisone and lenalidomide, with90% survival at 2 years[4]. Patients over 65 years old and those with significant concurrent illness can only receive the thirdtreatment, but these drugs have significant side effects, and the treatment effect is not obvious. To identify novel therapeutic options for the treatment of multiple myelomascientists are investigating the multi-scale pathogenesis of multiple myelomaat the intracellular, intercellular and tissue scalesand employing molecular drugs to treat MMs.

Overall, 80–90% of myeloma patients develop bone lesions during their disease course [1]. Multiple myeloma bone disease is characterized by dysfunction of both OB-mediated bone formation and OC-mediated bone resorption [5]. Bone homeostasis is maintained by the balance between the synthesis of new bone by OBs and the removal of old bone by OCs. In MM, there is an imbalance in the proportion of OCs and OBs. OB activity is markedly decreased or absent, and OC bone resorption is activated[5,6]. In this study, the balance is defined by two standards: one, the ratio of OCs to OBs; and two, the absolute difference in the number of OCs and OBs within a reasonable interval.

**Fig 1:**
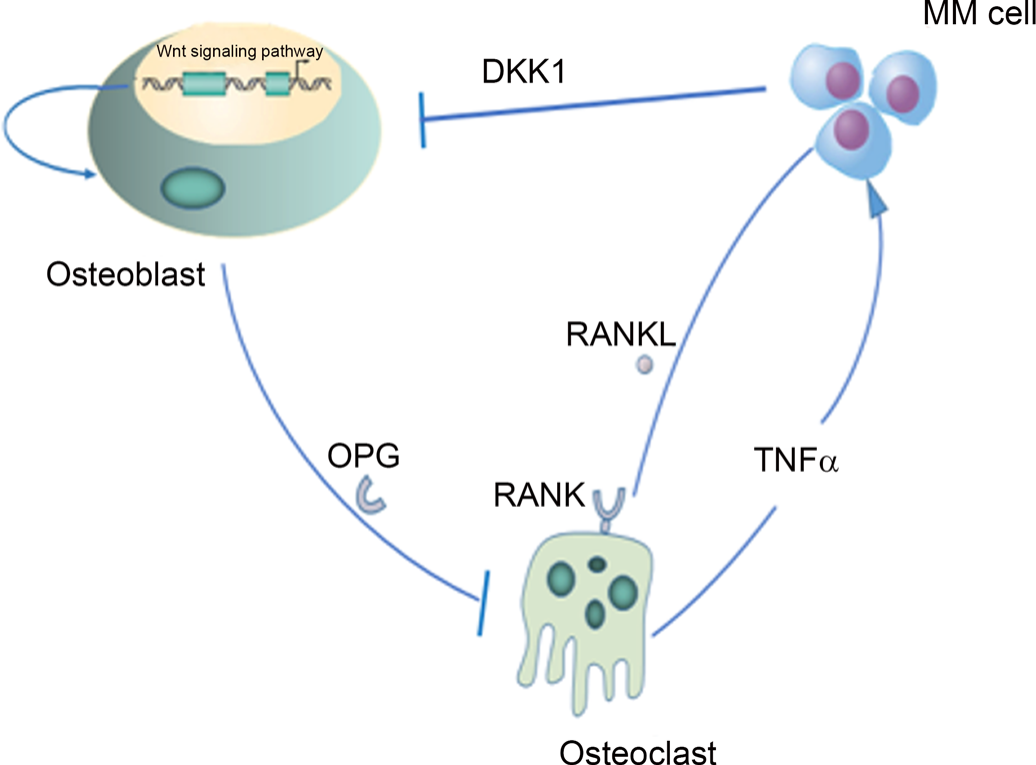
The signaling pathway for MMs, OBs and OCs. **I**: The Wnt signaling pathway stimulates the growth, differentiation and activity of osteoblasts. **II:**Dickkopf (DKK1) inhibits the phosphorylation of beta-catenin to prevent its degradation. **III:** The ratio of OPG/RANKL is negatively related to the number of OCs. **IV:** TNFα stimulates the formation of MMs and induces stromal cells to secrete factors, such as RANKL, that drive OC formation.

Multiple interactions in the myeloma bone marrow microenvironment are responsible for myeloma bone disease. A recent study[7] demonstrated that the DKK1-Wnt-OPG/RANKL intracellular signaling pathway can mediate the balance between OBs and OCs, which has becomeone of the most important factors in the pathogenesis of multiple myeloma. There are four major scenarios for the multi-scale pathogenesis of multiple myeloma (**Fig 1)**. **I:** The Wnt signaling pathway stimulates the growth, differentiation and activity of osteoblasts[8]. **II:** Dickkopf (DKK1) is secreted by MMs. Because DKK1 is a Wnt inhibitor, it inhibits the phosphorylation of beta-catenin to prevent its degradation [1]. Higher DKK1 expression has been found in myeloma patients and has shown a positive correlation with the advanced stages of myeloma [9]. **III:** DKK1 directly increasesRANKL and decreasesosteoprotegerin(OPG) expression in OBs[10]. The ratio of OPG/RANKL is negatively related to the number of OCs. **IV:** OCsproduce TNFα, which directly stimulates the formation of MMs and induces stromal cells to secrete factors, such as RANKL, that drive OC formation. TNFα is a potent inducer of OCs that blocks OB differentiation and promotes MM growth. MMs inhibit the growth of OBs and stimulate OCs to evoke a vicious cycle that promotes the imbalance between these two cell types. If OB formation is simultaneously inhibited by scenarios I and II and the growth of MMs is stimulated by scenario IV, the ratio of OPG/RANKL will decrease markedly, thereby escalating the generation of OCs[11].

Based on the pathogenesis of MM, several drugs have been developed to treat this disease[12]. Glucocorticoidshave been used for hematological cancer therapy, but they are associated with multiple adverse outcomes, such as the suppression of OCs and OBs[13]. BHQ880inhibits DKK1 and thus promotes bone formation, which in turn inhibits myeloma-induced osteolytic disease and MMgrowth[14]. Lidamycin accelerates the apoptosis of MMs.

However, most of these drugs do not workas well *in vivo* as they do *in vitro* due to absorption, distribution, metabolism, and toxicity (ADME) problems. For this reason, we employed multiple drugs to treat MM with an optimal drug combination plan to solve ADME problems and increase *in vivo* drug efficacy. However, it is impractical to quantitatively evaluate the optimaldrug combination *in vivo*. Hence, we employed a 3D multi-scale agent-based model that encompasses the intracellular, intercellular and tissue scales to address the following three specific aims: to develop a platform to describe the relationships among MMs, OBs and OCs; to investigate the pathogenesis of MM using this platform; and to employ this platform to identify the optimal drug combination for MM treatment.

The simulated results demonstrated that our model can be used not only to simulate the proliferation, migration and death of OBs, OCs and MMs but also to investigate the optimal use of these three drugs to inhibit MM growth and restore the balance between OCs and OBs.

## Materials and Methods

To describe tumor growth with the imbalance between osteoblasts and osteoclasts and to study the response of multiple myeloma to particular drug combinations, three types of agents are denoted in the model: MMs, OCs and OBs.Our multi-scale model consists of three biological scales: intracellular, intercellular and tissue. The intracellular scale describes the fundamental mechanisms for cellular phenotypic switches, and the intercellular scale bridges the tissue and intracellular scales as follows: (a) cytokines and drugs are delivered to the tumor microenvironment at the tissue scale; (b) MMs, OBs and OCs undergo phenotypic switching in response to stimulation by specific cytokines at the intercellular scale; and (c) DKK1 secreted by MMs affects the balance between OBs and OCs as well as their migration in response to secreted cytokines and drugs at the tissue scale. A 100*100*100 three-dimensional cube with four sub-compartments was employed to represent a slice of the virtual tumor extracellular matrix (ECM). The lattice size is 5 μm, which is approximately the same as the radius of a MM. Approximately one hundred MMs, OBs and OCs were mixed and initialized in the center of the lattice, forming a sphere.The age of the MMs, OBs and OCs was randomly initialized between 0 and 24 hours.

### Intracellular Scale: Phenotype switching of tumor cells as "agents"

**Fig 2:**
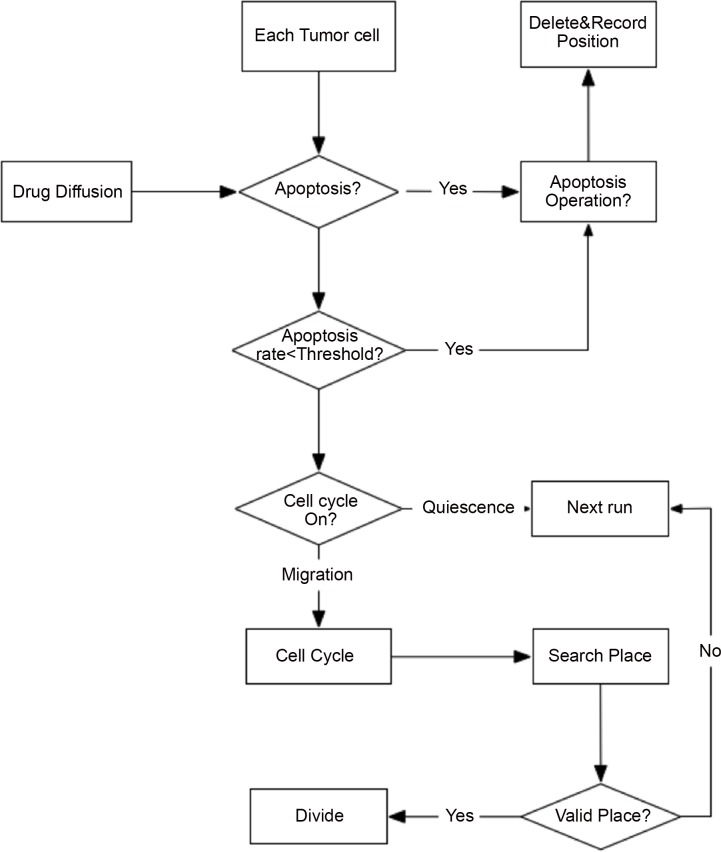
A flow chart of cellular phenotype switching at the intracellular scale. **Apoptosis:** At each time step, if the apoptosis probability of the cell (OBs, OCs or MMs) is less than the threshold, *Apop*_*rate*_, the cell will initiate apoptosis. **Proliferation**: The proliferation of each cell is influence by cytokines from other cells. **Migration:** A proliferating cell will migrate in the first three phases of cell cycle (G0/G1, S and G2), whereas it will look for an empty location to divide after entering the mitotic M phase.**Quiescence:** There are two possibilities for the cell to be in a quiescent state: the cell cannot go through the cell cycle, or the cell cannot find an appropriate free location for division.

At every simulation step (*Δt* = 2 *hours*), each MM, osteoclast orosteoblast determines its phenotype according to the following rules (**Fig 2)**.

#### Apoptosis

At each time step, if the apoptosis probability of the cell (OBs, OCs or MMs) is less than the threshold, *Apop*_*rate*_ the cell will initiate apoptosis. Any given cell takes 10 time steps to complete apoptosis and is then absorbed. As reported in previous studies [15], the cell’s apoptosis rate is defined according to an exponential distribution (**Eq 1)**:

$$Apop_{pro}=1-{\text{e}}^{-\lambda △\text{t}}$$ (1)

where △t denotes the time step(which is two hours), andλ is a positive number representing the average apoptosis frequency of each cell type.

**Eq 2** describes the impact of Lidamycin on the apoptosis rate of MMs based on the Hill function [16]:

$$\lambda^{mm}=\lambda_{0}{}^{mm}+\beta_{\text{mm}}{}^{L}\times \frac{{\left(\frac{L_{ijk}}{K_{L}{}^{mm}}\right)}^{2}}{1+{\left(\frac{L_{ijk}}{K_{L}{}^{mm}}\right)}^{2}}$$ (2)

where *L*_*ijk*_ denotes the relative Lidamycin concentration at the grid point (i, j, k), β_mm_^*L*^ is the maximum increase in the apoptosis rate, λ_0_^mm^ is the basal apoptosis rate of MMs, and (*K*_*L*_^*mm*^) is the threshold of Lidamycin that promotes MM apoptosis.

To restore the balance of OCs and OBs, the apoptosis rates of OCs and OBs should be regulated by drugs. **Eq 3** describes how glucocorticoids (GCs) promote the apoptosis of OBs and OCs based on the Hill function[17]:

$$\lambda^{n}=\lambda_{0}{}^{n}+\beta_{\text{n}}{}^{GC}\times \frac{{\left(\frac{G_{ijk}}{K_{GC}{}^{n}}\right)}^{2}}{1+{\left(\frac{G_{ijk}}{K_{GC}{}^{n}}\right)}^{2}}\;n=1,\;2$$ (3)

where n = 1 for OBs, n = 2 for OCs, *G*_*ijk*_ denotes the relative GC concentration at the grid point (*i*, *j*, *k*), and *K*_*GC*_^*n*^ is the threshold at which GCs promote the apoptosis of OCs and OBs.

#### Proliferation

**Eq 4** describes the proliferation probability of multiple myeloma cells based on a Hill function [18]:

$$P_{prol}{}^{\text{MM}}=P_{0}{}^{MM}+\beta_{\text{MM}}{}^{T}\times \frac{{\left(\frac{T_{ijk}}{K_{T}{}^{MM}}\right)}^{2}}{1+{\left(\frac{T_{ijk}}{K_{T}{}^{MM}}\right)}^{2}}$$ (4)

where *T*_*ijk*_ denotes the relative TNFα concentration at the grid point (*i*, *j*, *k*), *K*_*T*_^*MM*^ is the threshold at which TNFα promotes the proliferation of MMs, *P*_*prol*_^MM^ represents the impact of TNFαon the proliferation rate of MMs, *P*_0_^*MM*^ denotes the basal proliferation rate, and β_MM_^*T*^ is eamum level of proliferation of MMs.

**Eq 5** describes the proliferation probability of OBs based on the Hill function[19]:

$$P_{prol}{}^{ob}=P_{0}{}^{ob}+\beta_{\text{ob}}\times \frac{{\left(\frac{EWnt_{ijk}}{K_{W}}\right)}^{2}}{1+{\left(\frac{EWnt_{ijk}}{K_{W}}\right)}^{2}}$$ (5)

where *P*_*prol*_^*ob*^ represents the proliferation rate of OBs after responding to the positive effects of Wnt signaling, *P*_0_^*ob*^ denotes the basal proliferation rate, and β_ob_ is the maximum proliferation level.

**Eq 6** describes the proliferation probability of osteoclasts[20]:

$$P_{prol}{}^{OC}=P_{0}{}^{OC}+\frac{P_{pathway}{}^{OC}}{1+{\left(\frac{O\_RL_{ijk}}{K_{O\_RL}}\right)}^{2}}$$ (6)

where *P*_*prol*_^*oc*^ is the proliferation probability of osteoclasts, *P*_0_^*oc*^ denotes the basal proliferation probability, *p*_*pathway*_^*oc*^ is the maximum inhibition in relation to *O*_*RL*_*ijk*_, *K*_*O*_*RL*_ is the threshold of *O*_*RL*_*ijk*_ that inhibits the proliferation of osteoclasts, and *O*_*RL*_*ijk*_ denotes the concentration ratio of OPG to RANKL at the grid point (*i*, *j*, *k*).

**Eq 7** utilizes a Bernoulli function to determine whether the cell enters the cell cycle:

$$\{\begin{array}{l} \;{\text{C}}_{\text{rand}}\in \left[0,{\text{p}}_{\text{prol}}\right)\;\text{cell cycle ON}\\ {\text{C}}_{\text{rand}}\in \left[{\text{p}}_{\text{prol}},1\right)\;\text{cell cycle OFF} \end{array}$$ (7)

If C_rand_ falls in the interval [0,p_prol_), the cell enters the cell cycle and starts to proliferate; otherwise, the cell stays quiescent and awaits the next round. In the M phase of the cell cycle, a cell will divide if it finds at least one free location within the search distance.

#### Migration

Non-proliferating cells will migrate at each step. Proliferating cells will migratein the first three phases of the cell cycle (G0/G1, S and G2), whereas they will look for an empty location to divide after entering the mitotic M phase [21]. This will be discussed in detail in the next section.

#### Quiescence

There are two possibilities for the cell to be in a quiescent state: one, the cell cannot go through the cell cycle as a result of **Eq 5**; and two, the cell cannot find an appropriate free location for cell division.

### Intercellular Scale: The dynamics of molecules in signaling pathways for each cell and the rules for choosing the “most attractive” location

*EWnt*_*ijk*_ is the impact of DKK1 on Wnt as described by **Eq 8**:

$$EWnt_{ijk}=E_{0}+\frac{\beta_{\text{w}}}{1+{\left(\frac{D_{ijk}}{K_{D}}\right)}^{2}}$$ (8)

where *D*_*ijk*_ denotes the relative DKK1 concentration at the grid point (*ijk*), K_D_ is the threshold of DKK1 that inhibitsWnt signaling, *EWnt*_*ijk*_ represents the impact of DKK1 on Wnt signaling, and E_0_ and β_w_ are the basal and maximum inhibition levels in relation to Wnt signaling.

As previously reported[22], an imbalance between OBs and OCs results when OCs outnumber OBs in the context of MM. BHQ880 inhibits DKK1 to reduce Wnt signaling, and **Eq 8** is replaced by **Eq 9**:

$$EWnt_{ijk}=E_{0}+\frac{\beta_{\text{w}}}{1+{\left(\frac{D_{ijk}{}^{e}}{K_{D}}\right)}^{2}}$$ (9)

where ${D_{ijk}}^{e}=\frac{{D_{ijk}}^{2}}{D_{ijk}+B}$, represents the current effective DKK1 concentration at the grid point (i, j, k), and B denotes the dose of BHQ880.

Each living MM chooses the “most attractive” location to proliferate or migrate based on the following rules:

1) A non-M phase cell at position *p*_0_ will always search for a position with greater nutrition to migrate or divide. The six nearest (Moore) neighbors of *p*_0_ are the candidate locations. Each one is ranked by **Eq 10:**

$$R_{l}=\frac{1}{4}p\left(r_{l}\right)\cdot V_{l}$$ (10)

where *R*_*l*_ is the ranking score of each candidate position *p*_*ijk*_^*l*^, *r*_*l*_ is the distance from the candidate location *p*_*ijk*_ to position *p*_0_, and *p*(*r*_*l*_) is the probability that the cell moves to the candidate location *p*_*ijk*_.

$$p\left(r_{l}\right)=\frac{1}{4\pi D\Delta t}\cdot \text{exp}\left(\frac{-r_{l}{}^{2}}{4\pi D\Delta t}\right)$$ (11)

where *D* is the distance squared over time.

MMs try to avoid isolated or over-crowded locations, but they are not as sensitive as solid tumor cells.The preference for neighborhoods (*V*_*l*_) is denoted by **Eq 12**:

$$V_{l}=\{\begin{array}{l} 1/8\;P_{ijk}\;\text{has}\;5\sim 6\;\text{neighbor cells}\\ 1/4\;P_{ijk}\;\text{has}\;3\sim 4\;\text{neighbor cells}\\ 1\;P_{ijk}\;\text{has}\;1\sim 2\;\text{neighbor cells}\\ 1/16\;P_{ijk}\;\text{has no neighbor cells} \end{array}$$ (12)

The ranks of the candidates are normalized in **Eq 13**:

$${\tilde{R}}_{l}=\frac{R_{l}}{\sum_{l}R_{l}}$$ (13)

To facilitate die-casting, all the normalized ranks are incorporated to form a scale *S* in **Eq 14**, in which each candidate corresponds to a range *S*_*l*_

$$S=\{S_{l}:S_{l}=[\sum_{m=0}^{m=(l-1)}{\tilde{R}}_{m},{\tilde{R}}_{l}+\sum_{m=0}^{m=(l-1)}{\tilde{R}}_{m})]$$ (14)

*S* is an ordered set of *S*_*l*_. Each *S*_*l*_is a region in the interval [0,1] and relates to the *lth*candidate site. Die rolling generates a random value *d* ∈ [0,1). If *d* falls in *S*_*l*_, the corresponding candidate location $\tilde{R_{l}}$ will be chosen as the next migration or proliferation site.

2) If no space is available, the cell will become reversiblyquiescent and will await the next round.

### Tissue Scale: cytokine and drug diffusion

**Eq 15** describes cytokine (DKK1, RANKL, OPG, and TNF-α) diffusion in the 3D extracellular matrix.

$$C_{ijk}\left(t+1\right)=\left\{D_{ijk}\left(t\right)\times \left(1-\lambda_{C}\right)+\frac{\lambda_{C}}{6}\times \sum_{l=1}^{6}C_{ijk}^{l}\left(t\right)+\chi_{mm}\left(t,P_{ijk}\right)*Se_{G}\right\}*\left(1-DEG\right)$$ (15)

*C*_*ijk*_(*t* + 1) is the cytokine value at the location *P*_*ijk*_ in the *t* + 1 time step, *λ*_*C*_ is the diffusivity, and $C_{ijk}^{l}(t),l=1,2,\ldots ,6$ are the cytokine values of *P*_*ijk*_’s six nearest neighbors in the current time step. If the cytokine is DKK1 or RANKL and there is aMM at *P*_*ijk*_, the time-dependent characteristic function *χ*_*mm*_ (*t*, *P*_*ijk*_) is set to 1; otherwise, this value is set to 0.If the cytokine is OPG and there is an OB at *P*_*ijk*_, the time-dependent characteristic function *χ*_*mm*_ (*t*, *P*_*ijk*_) is set to 1; otherwise, this value is set to 0.If the cytokine is TNF-α and there is an OC at *P*_*ijk*_, the time-dependent characteristic function *χ*_*mm*_ (*t*, *P*_*ijk*_) is set to 1; otherwise, this value is set to 0. ***Se***_***G***_ and *DEG* represent the cytokine secretion rate andthe degradation rate, respectively.

As discussed previously, BHQ880, GCs and Lidamycin were utilized in our model to restore the balance between OCs and OBs. Additionally, we developed this novel using *in silico* algorithms to identify the optimum multi-drug combination to restore this balance. BHQ880 regulates the proliferation of OBs and OCs by inhibiting DKK1 [23]. GCs promote the apoptosis of both OBs and OCs. Because MMs play an important role in generating an imbalance between OBs and OCs by secreting DKK1, Lidamycin can restore the balance by increasing the apoptosis of MMs [24]. **Eq 16** describes how these multiple drugs diffuse into the surrounding tissue before being taken upby MMs, OBs and OCs.

$$DR_{ijk}\left(t+1\right)=\{DR_{ijk}\left(t\right)\times \left(1-\lambda_{d}\right)+\lambda_{d}/6\times \sum_{l=1}^{6}DR_{ijk}^{l}\left(t\right)+\chi_{oc}\left(t,P_{ijk}\right)*Pe_{d}\}*\left(1-U_{d}\right)$$ (16)

where *DR*_*ijk*_ (*t* + 1) represents the concentration of any one of these three drugs at the location *P*_*ijk*_ in the (t+1) time step, and *λ*_*d*_is the drug diffusivity. ${DR}_{ijk}^{l}(t),\;l=1,2,\ldots ,6$ are the drug concentrations of *P*_*ijk*_’snearest six neighbors in the current time step. *Pe*_*d*_ is the vessel permeability of the drug, and *U*_*d*_ represents the drug uptake rate.

**Fig 3:**
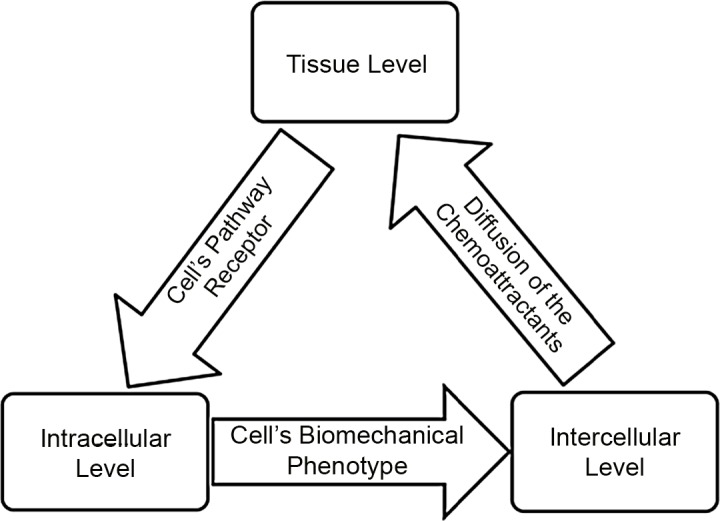
Schematic of the multi-scale modeling of OBs, OCs and MMs. **Intracellular scale**: describes the communication among myeloma cells, osteoclasts and osteoblasts and their ‘phenotypic’ switches. **Intercellular scale**: describes the dynamics of molecules in signaling pathways for each cell after receiving cytokine stimulation from other cells and the specific migration rules for cells. **Tissue scale**: describes the diffusion of drugs and cytokines.

**Fig 3** shows a flowchart of the algorithm.At the intracellular scale, **Eqs 1–7** describe the phenotypic (migration, proliferation, quiescence or apoptosis) switches of the MMs, OBs and OCs. At the intercellular scale,the dynamics of molecules in the cellular signaling pathways after receiving cytokine stimulation from other cellsare represented by **Eqs 8 and 9**. In addition, MMs compete for the best location to migrate or proliferate, as defined by the distance to candidate locations and the cell density(**Eqs 10–14).** At the tissue scale, a set of simplified reaction–diffusion equations (**Eqs 15 and 16**) describe the spatial concentration changes in cytokines(DKK1, OPG, RANKL, and TNF-α) and drugs(BHQ880, Lidamycin, and glucocorticoids). These changes not only remodel the tumor microenvironment but also greatly influence the behavior of MMs, OCs and OBs (cytokine secretion, proliferation, migration, or apoptosis) at the intracellular scale. The details of the model parameters are listed in **Table 1**.

**Table 1:** System parameters.

**Symbol**	**Variable**	**Initial value**
*E*_0_	Basic inhibition level of Wnt	0
*β*_*W*_	Maximum inhibition level of Wnt	0.5
*K*_*D*_	Threshold of DKK1 that inhibitsWnt	1
*P*_0_^*o*^	Initial proliferation rate of osteoblasts	0.1
*β*_*ob*_	Maximum stimulation of OBs	1
*K*_*W*_	Threshold of Wntthat activates OBs	0.8
*P*_0_^*oc*^	Initial proliferation rate of osteoclasts	0.1
*K*_*O*:*RL*_	Threshold of OPG:RANKLthat inhibits the proliferation of OCs	1
*P*_0_^*mm*^	Initial proliferation rate of MMs	0.15
β_mm_^*L*^	Maximum activation ofMM proliferation	0.5
*K*_*T*_^*mm*^	Threshold of TNF-α that promotesMM proliferation	1
λ_0_^*mm*^	Initial apoptosis rate of MMs	0
β_mm_^*T*^	Maximum increase in the apoptosis of MMs	0.05
*K*_*L*_^*mm*^	Threshold of Lidamycinthat activates MM	1.00E-07
λ_0_^1^	Initial apoptosis rate of osteoblasts	3.50E-02
β_1_^*GC*^	Level of GCsthat impactsOB apoptosis	0.15
*K*_*GC*_^1^	Threshold of GCs that promotes OB apoptosis	0.15
λ_0_^2^	Initial apoptosis rate of osteoclasts	0
*K*_*GC*_^2^	Threshold of GCsthat promotes OC apoptosis	1.50E-06
*β*_2_^*GC*^	Initial apoptosis rate of osteoclasts	0

## Results

**Fig 4:**
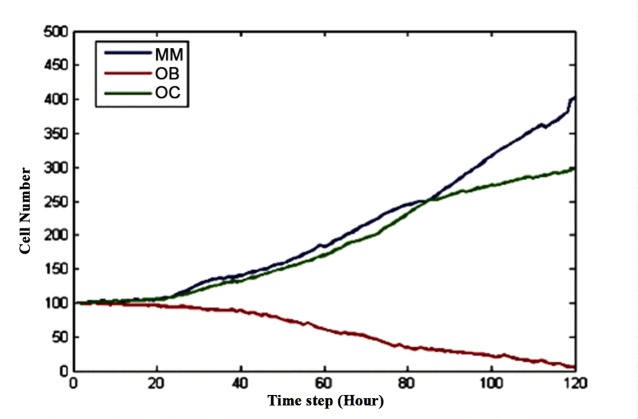
The dynamics of OBs, OCs and MMs without drug treatment.

This model was implemented using Visual C++ [25] and was used to predict the response of multiple myeloma cells to drug combinationsas well as the related balance between osteoclasts and osteoblasts in response to treatment with drug combinations. **Tissue scale behavior**: The simulation time encompassed time steps 0 to 120 and each time step represented 2 hours. **Fig 4** shows the dynamics of three types of cells (MMs, OBs and OCs). Note that a unique color denotes each cell type: osteoclast (green), osteoblast (red), and multiple myeloma cell (blue).

**Fig 5:**
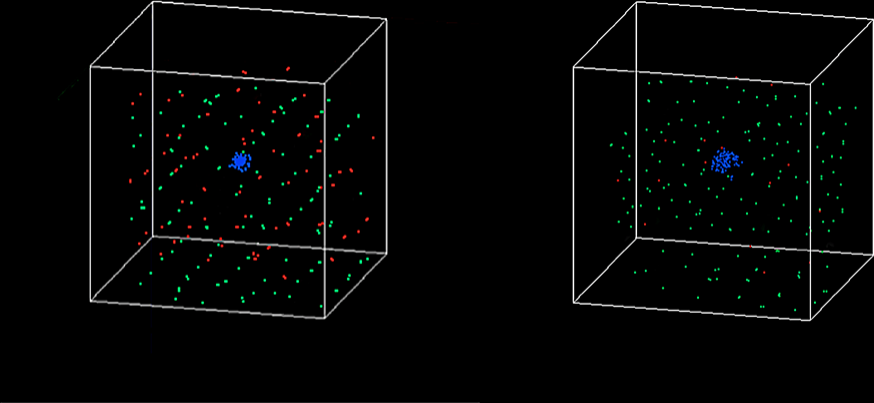
3D snapshots of the tumor system without drug treatment at (a) time step 90 and (b) time step 120.

**Fig 4** shows that the three curves began to separate at time step 20. The number of MMs (blue) intersected the number of OCs (green) around time step 90. After 120 time steps, the number of OCs and MMs increased three-fold and four-fold, respectively, compared to their initial values, whereas the number of OBs approached zero. **Fig 5** shows the three-dimensional snapshots of the OBs, OCs and MMs at time points 0 and 120 without drug treatment.

**Fig 6:**
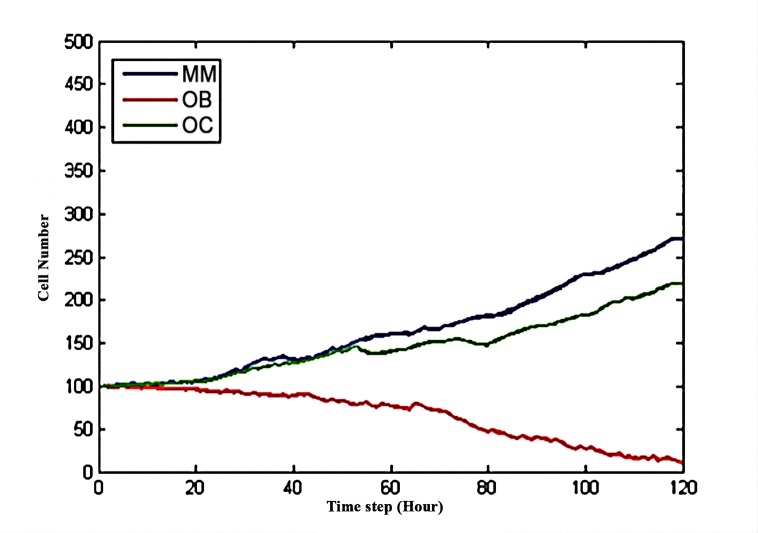
The effect of BHQ880 on the number of OBs, OCs and MMs.

**Fig 7:**
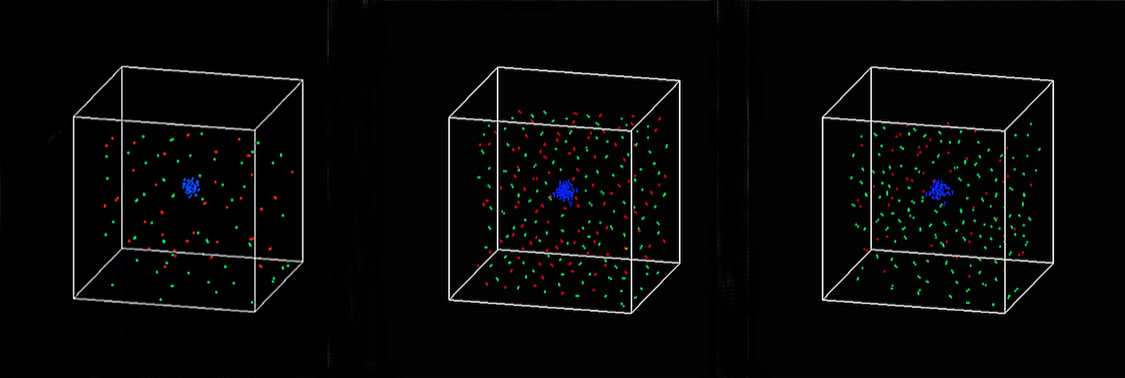
3D snapshots of the tumor system with single-agent BHQ880 treatment at (a) time step 40, (b) time step 50 and (c) time step 120.

BHQ880 was injected at time step 20 to restore the balance between OBs and OCs, when the populations of the three cell types began to differ, asshown in **Fig 4**. BHQ880 can directly increase the proliferation rates of MMs and OCs and decrease the proliferation rate of OBs by regulating the function of DKK1. **Fig 6** shows that BHQ880 markedly decreased the number of MMs and OCs while increasing the number of OBs compared to the results reported in **Fig 4**; however, this drug could not change the trend of the three curves. **Fig 7** shows the 3D snapshots of the tumor system with the single-agent BHQ880 treatment at the following time steps: t = 40, t = 50 and t = 120.

**Fig 8:**
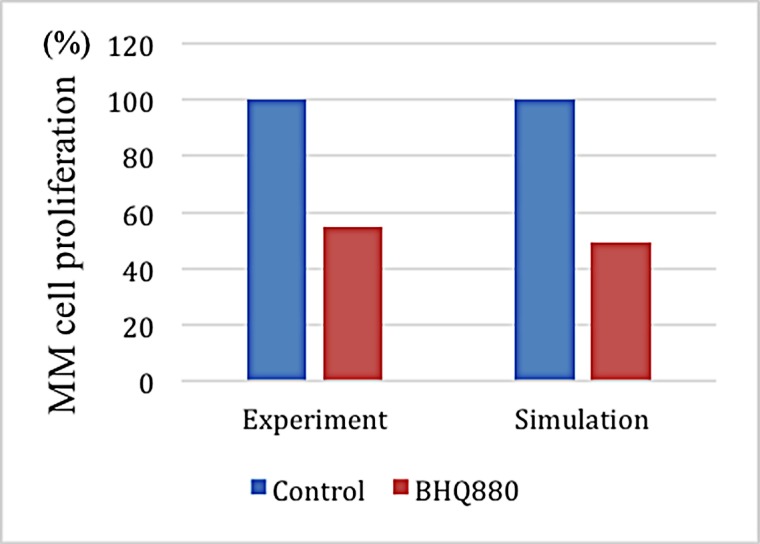
A comparison of the experimental and simulated data after BHQ880 treatment.

The MM proliferation rate served as the target to verify the predictive power of the model after injecting BHQ880. **Fig 8** shows a small difference between the simulated and experimental data [16] after 120 time steps under similar initial conditions.

**Fig 9:**
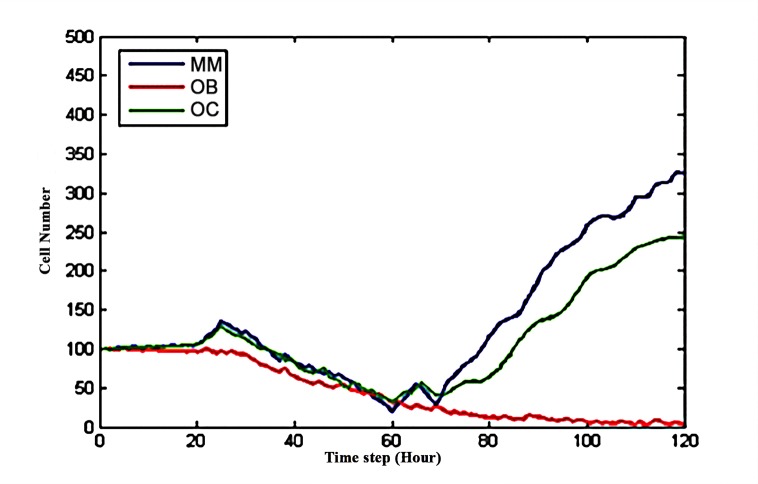
The effect of GCs on the number of OBs, OCs and MMs.

**Fig 10:**
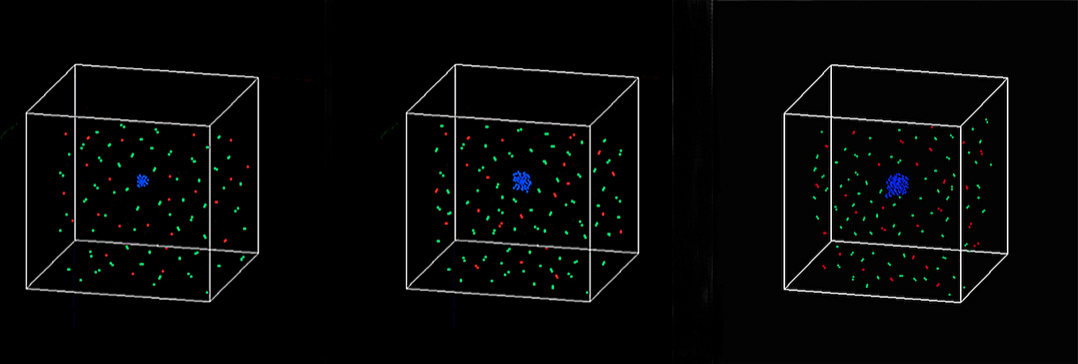
3D snapshots of the tumor system with single-agent GC treatment at (a) time step 60, (b) time step 65 and (c) time step 120.

**Fig 11:**
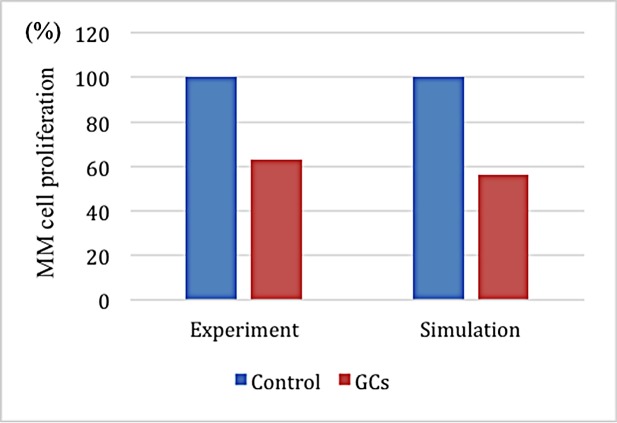
A comparison of the experimental[17] and simulated data after GC treatment.

GCswere employed to restore the balance by increasing the apoptosis rates of OCs and OBs. **Fig 9** shows that when GCs were injected at time step 20, the populations of all three celltypes quickly decreased before time step 60. Thereafter, the MM and OC populations quickly increased, whereas the number of OBs decreased to nearly zero during the rest of the simulation. This phenomenon is also illustrated in **Figs 4** and **6**. **Fig 10** shows the spatial information of these three cell types at time steps t = 60, t = 65 and t = 120. The MM proliferation rate served as the target to verify the predictive power of the model after the injection of GCs. **Fig 11** shows a small difference between the simulated and experimental data [26] after 120 time steps undersimilar initial conditions.

**Fig 12:**
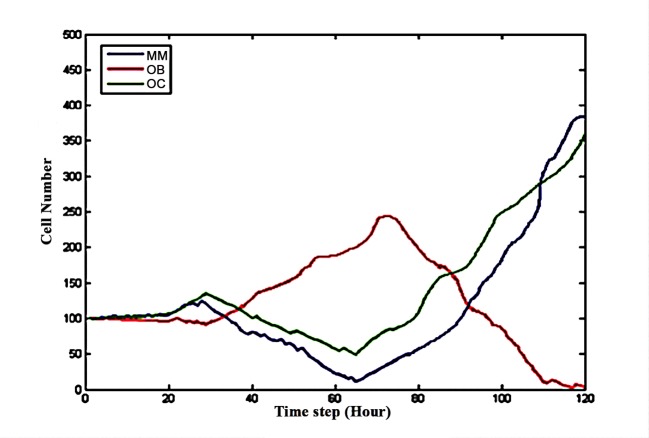
The effect of Lidamycin on the number of OBs, OCs and MMs.

**Fig 13:**
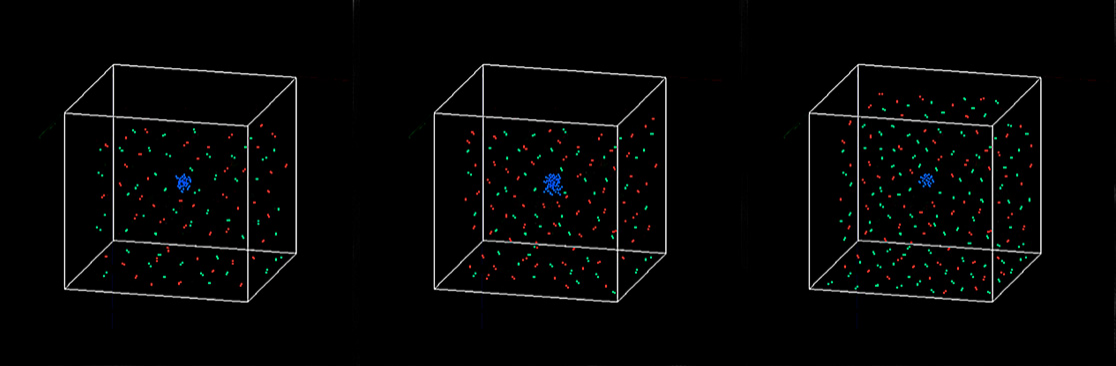
3D snapshots of the tumor system with single-agent Lidamycin treatment at (a) time step 65, (b) time step 70 and (c) time step 85.

**Fig 14:**
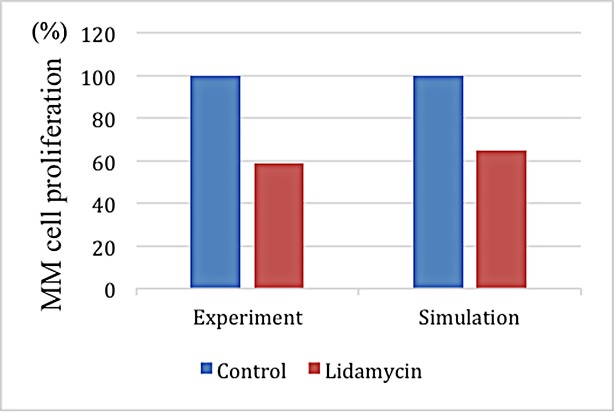
A comparison of the experimental and simulated data after Lidamycin treatment.

Lidamycin promotes the apoptosis of MMs. **Fig 12** shows that the number of MMs and OCs markedly decreased before time step 65, whereas the number of OBs significantly increased before time step 70. After time step 65, the MMand OC populationsconsiderably increased. Around time step 85, the OB and OC populations intersected. After time step 120, the number of OBs decreased to almost zero. In particular, the final number of MMs and OCs after treatment with Lidamycin was greater than that without treatment (**Fig 4**). **Fig 13** shows the spatial information of these three cell types at time steps65, 70 and 85.TheMM proliferation rate is employed as the target to verify the predictive power of the model after GC injection. **Fig 14** shows a small difference between the simulated and experimental data [18] after 120 time steps undersimilar initial conditions.

**Fig 15:**
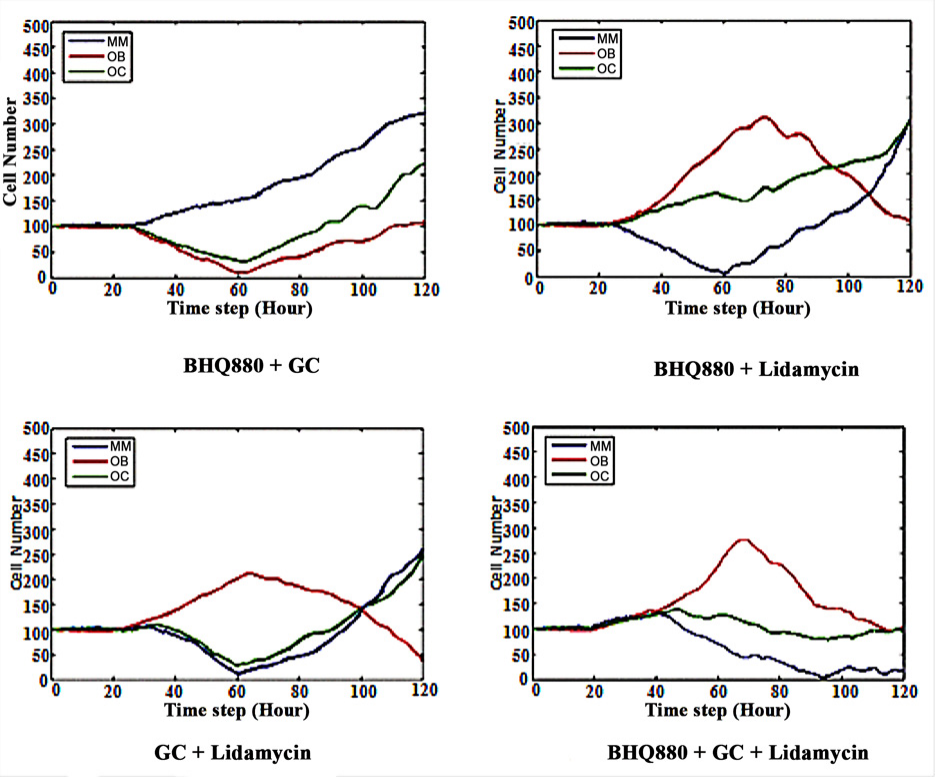
The effect of BHQ880 + GCs, BHQ880 + Lidamycin,GCs + Lidamycin, and BHQ880 + GCs + Lidamycin on the number of OBs, OCs and MMs.

**Figs 6–14** illustrates that a single drug neither restored the balance nor killed all the MMs. Hence, multi-drug combination treatment plans were developed. Each combination of two or three drugs was injected into the patient between time steps t = 20 andt = 60, and we observed the treatment effect until time step t = 120. Similar to previous research [17], the ratio of these drugs was 1:1. The diagram of the upper left corner in **Fig 15** shows that the growth rates of both OCs and OBs were greatly inhibited by two drugs, whereas the MM population increased from time step t = 20 to t = 60.

The diagram of the upper right corner in **Fig 15** demonstrates that the combination of BHQ880 and Lidamycin markedly inhibited MMs between time steps 20 and 60 while increasing the number of OBs, but this combination did not affect OCs. After time step 60, the number of MMs quickly increased, whereas the number of OBs slowly increased initially and began to decrease around time step 75.

The diagram of the lower left corner in **Fig 15** shows that the combination of GCs and Lidamycin clearly regulated MMs and OCs between time step 20 and 60 and increased the number of OBs. However, without these drugs, the number of MMs and OCs quickly increased, whereas the number of OBs decreased.

The diagram of the lower right corner in **Fig 15** shows that the combination of GCs, BHQ880 and Lidamycin not only significantly regulated MMs and OCs between time step 20 and 60 but also increased the number of OBs. After discontinuing the drug (time step 60), the number of MMscontinued to decrease, and the balance between OCs and OBs was restored. Because one of the major aims of this study was to investigate which drug combination efficiently restores the initial balance between OCs and OBs and kills MMs to avoid relapse, it was necessary to develop **Eq 17** to evaluate the performance of the multi-drug combinations.

$$R_{drug}=N_{MM}^{120}\times \left|N_{OC}^{120}-N_{OC}^{0}\right|\times \left|N_{OB}^{120}-N_{OB}^{0}\right|$$ (17)

$N_{MM}^{120}$, $N_{OC}^{120}$, and $N_{OB}^{120}$ are the numbers of MMs, OCs and OBs, respectively,at time step t = 120. $N_{OC}^{0}$ and $N_{OB}^{0}$ are the initial numbers of OCs and OBs, respectively.

Our simulated results (**Figs 6–15** and **Table 2**) indicate that neither single drug nor two-drug combinations could restore the initial balance between OBs and OCs and avoid relapse. Nevertheless, the three-drug combination (**Fig 15**) showed significant potential for realizing this aim. For this reason, we ascertained which combination of these three drugs had the best performance as measured by the smallest *R*_*drug*_ in **Eq 17**.

**Table 2:** Response of R_drug_ to different drug combinations.

Drug therapy	*R*_*drug*_
BHQ880	2.2 E+06
GCs	4.6 E+06
Lidamycin	9.2 E+06
BHQ880/GCs	8.1 E+04
BHQ880/Lidamycin	1.8 E+05
GCs/Lidamycin	2.0 E+06
BHQ880/GCs/Lidamycin	8.0 E+01

### Cytokine analysis in response to combination therapy

**Fig 16:**
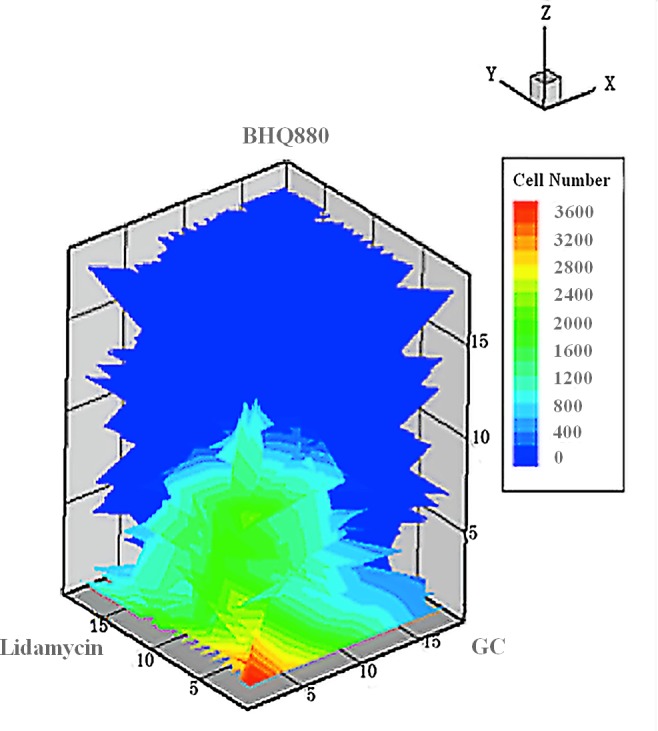
The effects of BHQ880, GCs and Lidamycinon restoring the balance between OCs and OBs and killing MMs.

In total, 20 doses of the three drugs were evaluated, from 0.1X to 10X in geometric sequence relative to the original dose. Then, we explored the efficacy of various combinations of the three drugs. Although conventional and high-dose chemotherapy can evoke frequent responses in patients with multiple myeloma, it remains an incurable disease, largely due to drug resistance. The bone marrow microenvironment not only promotes the survival and growth of myeloma cells but alsoparticipates in the development of resistance to single conventional and novel agents. To prevent recurrent multiple myeloma and induce complete remission, it is important to develop combination therapy strategies that inhibit certain interactions between myeloma cells and the bone marrow microenvironment [13,27]. ***R***_***drug***_ represents the drug efficacy. The minimum ***R***_***drug***_ value occurred at the doses of 10, 6, and 4 for the combination of GCs, Lidamycin and BHQ880(**Fig 16**).

**Fig 17:**
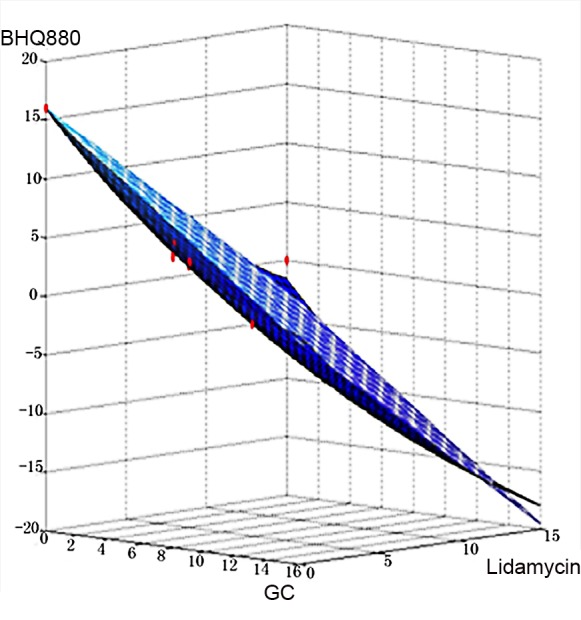
Synergy prediction of the combination of BHQ880, GCs and Lidamycin based on the Loewe combination index.

The Loewe additivity [28,29] evaluates whether the combination effect of BHQ880, GCs and Lidamycin is synergistic.The combination index of Loewe synergy is defined as the ratio of the total effective drug dose (combination versus single drugs) required to achieve a given effect, as stipulated in **Eq 18**:

$${\text{CI}}_{\text{Loewe}}=\frac{d_{1}}{{\text{GC}}_{x}^{\left(1\right)}}+\frac{d_{2}}{{\text{GC}}_{x}^{\left(2\right)}}+\frac{d_{3}}{{\text{GC}}_{x}^{\left(3\right)}}$$ (18)

where *d*_1_ (BHQ880), *d*_2_ (GC) and *d*_3_ (Lidamycin) are the drug combination doses located in the combination isobologram with respect to ***R***_***drug***_. ${GC}_{x}^{\left(1\right)}$, ${GC}_{x}^{\left(2\right)}$ and ${GC}_{x}^{\left(3\right)}$ represent the single-agent concentrations of BHQ880, GCs or Lidamycin with respect to ***R***_***drug***_, respectively. CI_Loewe_ < 1, CI_Loewe_ = 1 and CI_Loewe_ > 1 indicate Loewe synergy, antagonism and addition, respectively. In **Eq 18**, ***R***_***drug***_ = 960is the threshold for evaluating the drug effect. The simulated results show that the combination of BHQ880, GCs and Lidamycin is synergistic (**Fig 17**).

### Parameter sensitivity analysis

To evaluate the impact of the parameter values on the behavior of the multiple myeloma cancer modeling system, we analyzed the sensitivity of our model based on the following parameters: *β*_*ω*_, *β*_*mm*_^*T*^, *K*_*D*_, *K*_*w*_, *λ*_*C*_, *DEG*, *λ*_*d*_, *Se*_*G*_, *Pe*_*d*_, *and U*_*d*_ We varied each parameter individually over the ranges shown in **Table 1**, while fixing other parameters at their base values. The ranges of the parameters were obtained from the literature, and these values are listed in **Table 1**. We were limited by the relatively high computing cost of the ABM, so we performed 10 simulations for each set of parameters. To assess the influence of the parameters, we calculated the Spearman rank-order correlation [30]of each parameter versus the number of total MMs, OBs, and OCs. **Table 3** shows the Spearman rank-order correlation *ρ* and the *p* – *value* for each parameter. Additionally, this table explores the parameters that are closely related to the number of MMs, OBs and OCs: vessel permeability of thedrug (*Pe*_*d*_), the drug diffusion constant (*λ*_*d*_), and the drug uptake rate (*U*_*d*_). Because the threshold of Wntthat promotes OB proliferation (*K*_*w*_) and the threshold of DKK1 that inhibits Wnt(*K*_*D*_) are closely correlated with osteoblast cell number, it can be concluded that DKK1 and Wnt play an important role in osteogenesis.

**Table 3:** Spearman rank-order correlations and p-values for model parameters and simulation outcomes.

	Multiple myeloma cells		Osteoblasts		Osteoclasts	
parameters	Spearman *ρ*	*p* – *value*	Spearman *ρ*	*p* – *value*	Spearman *ρ*	*p* – *value*
***β***_***ω***_	0.1071	0.9549	0.1307	0.5409	0.1179	0.8192
***β***_***mm***_^***T***^	0.1839	0.4592	0.1459	0.6011	-0.3596	0.4367
***K***_***D***_	0.4116	0.0983	0.5206	5.3261 E-2	0.1216	0.6935
***K***_***w***_	-0.7195	0.0091	-0.9519	8.9112 E-7	-0.1659	0.7905
***λ***_***C***_	0.3552	0.1985	-0.0178	0.7561	0.3819	0.2969
***DEG***	-0.1268	0.6389	0.3145	0.2965	0.1181	0.7953
***λ***_***d***_	0.9816	9.3051 E-8	0.9761	6.3275 E-7	0.8995	1.6231 E-4
***Se***_***G***_	0.5545	0.0790	0.6230	0.091	0.6534	0.0596
***Pe***_***d***_	-.0.0901	1.1502	-0.0359	0.9535	0.6051	0.0926
***U***_***d***_	0.9609	8.4209 E-6	0.9872	5.6201 E-8	0.9910	1.9602 E-9

## Discussion

Serious bone disease frequentlyoccursin multiple myeloma patients. The altered bone microenvironment sustains the survival of MMs. Therefore, effectively treating myeloma requires targeting not only MMs but also the imbalance betweenOBs and OCs. The current study established a novel multi-scale agent-based 3D model that encompasses a multiple myeloma growth module to investigate the restoration of the balance between associated OCs and OBs.This research aimed to investigate the relationships among the growth of MMs, OCs and OBs as well as to identify the optimum synergistic drug combinations that restore the balance between OCs and OBs and kill MMs. We simulated the growth of OBs, OCs and MMs before and after drug treatment. During MM proliferation, the number of OCs increases, whereas the number of OBs decreases. This results in an uncoupling of osteoclasticresorption and osteoblastic bone formation. It is obvious that the high secretion of DKK1, the canonical Wnt inhibitor,by MMs impacts the OB-OC balance and induces MM growth [7].BHQ880 (a DKK1 antibody) promotes OB formation by inhibiting DKK1. Two other drugs (GCs and Lidamycin) regulate cellular apoptosis. As the results show, BHQ880 had no significant effect on inhibiting the growth of MMs or restoring the balance between OBs and OCs (**Figs 6–8**). Though GCs and Lidamycin initially reduced the growth rate of MMs, neither drug (**Figs 9–14**) individually restored the balance between OBs and OCs and avoided MM relapse. Furthermore, the cell cytotoxicity caused by GCs and Lidamycin decreases quickly during MM progression. Although high-dose chemotherapies (GCs and Lidamycin) can significantly increase the apoptosis of multiple myeloma cells, the disease inevitably relapses due to the acquisition of drug resistance. One of the reasons for drug resistance is that the bone marrow microenvironment is associated with the resistance to single-agent chemotherapy. Combination therapies will be required to increase cytotoxicity and overcome drug resistance. It has been reported that OCsinduce myeloma proliferation [31]. OB stimulation may also have beneficial effects on decreasing MMgrowth and bone destruction [32]. The OB-OC balance might play a crucial role in supporting myeloma relapse. Therefore, we consider that multi-drug combinations are necessaryto effectively inhibit MM growth and relapse. The simulated results demonstrate that two-drug combinations **(Fig 15**) offer a better treatment effect than a single drug (**Figs 6–14**), but MMs kept growing at the late stage of treatment due to the imbalance between OBs and OCs. Although two-drug combinations are not ideal for MM treatment, the results inspired us to evaluate three-drug combinations to treat MM. As shown in **Fig 15**, MM growth decreased, and the OB-OC balance was restored by a three-drug combination.To optimize the best ratio for the three-drug combination, **Eq 16** was developed to evaluate drug efficacy. **Fig 16** shows that a 1:2:4 ratioof BHQ880, GCs and Lidamycin is the best for killing MMs and restoring the balance. Lastly, **Fig 17** illustrates that the three-drug combination has the strongest synergistic effect.

In general, this research details how to employ a 3D multi-scale agent-based cancer model to describe the communication among MMs, OBs and OCs to the best of our knowledge, and it also investigates the optimal use of drug combination therapy to cure multiple myeloma and restore the balance between OBs and OCs. These simulated results demonstrate that restoring the balance of OBs and OCs not only markedly inhibits the growth rate of MMs but also ensures the stable metabolism of bone tissue.
